# Using Smartwatches to Observe Changes in Activity During Recovery From Critical Illness Following COVID-19 Critical Care Admission: 1-Year, Multicenter Observational Study

**DOI:** 10.2196/25494

**Published:** 2022-05-02

**Authors:** Alex Hunter, Todd Leckie, Oliver Coe, Benjamin Hardy, Daniel Fitzpatrick, Ana-Carolina Gonçalves, Mary-Kate Standing, Christina Koulouglioti, Alan Richardson, Luke Hodgson

**Affiliations:** 1 Department of Intensive Care Medicine Worthing Hospital University Hospitals Sussex National Health Service Trust Worthing United Kingdom; 2 School of Sport and Health Sciences University of Brighton Brighton United Kingdom; 3 Department of Intensive Care Medicine East Sussex National Health Service Trust Eastbourne United Kingdom; 4 School of Biosciences and Medicine University of Surrey Guildford United Kingdom

**Keywords:** COVID-19, telemedicine, rehabilitation, critical illness, smartphone, digital health, mobile health, remote therapy, device usability

## Abstract

**Background:**

As a sequela of the COVID-19 pandemic, a large cohort of critical illness survivors have had to recover in the context of ongoing societal restrictions.

**Objective:**

We aimed to use smartwatches (Fitbit Charge 3; Fitbit LLC) to assess changes in the step counts and heart rates of critical care survivors following hospital admission with COVID-19, use these devices within a remote multidisciplinary team (MDT) setting to support patient recovery, and report on our experiences with this.

**Methods:**

We conducted a prospective, multicenter observational trial in 8 UK critical care units. A total of 50 participants with moderate or severe lung injury resulting from confirmed COVID-19 were recruited at discharge from critical care and given a smartwatch (Fitbit Charge 3) between April and June 2020. The data collected included step counts and daily resting heart rates. A subgroup of the overall cohort at one site—the MDT site (n=19)—had their smartwatch data used to inform a regular MDT meeting. A patient feedback questionnaire and direct feedback from the MDT were used to report our experience. Participants who did not upload smartwatch data were excluded from analysis.

**Results:**

Of the 50 participants recruited, 35 (70%) used and uploaded data from their smartwatch during the 1-year period. At the MDT site, 74% (14/19) of smartwatch users uploaded smartwatch data, whereas 68% (21/31) of smartwatch users at the control sites uploaded smartwatch data. For the overall cohort, we recorded an increase in mean step count from 4359 (SD 3488) steps per day in the first month following discharge to 7914 (SD 4146) steps per day at 1 year (*P*=.003). The mean resting heart rate decreased from 79 (SD 7) beats per minute in the first month to 69 (SD 4) beats per minute at 1 year following discharge (*P*<.001). The MDT subgroup’s mean step count increased more than that of the control group (176% increase vs 42% increase, respectively; +5474 steps vs +2181 steps, respectively; *P*=.04) over 1 year. Further, 71% (10/14) of smartwatch users at the MDT site and 48% (10/21) of those at the control sites strongly agreed that their Fitbit motivated them to recover, and 86% (12/14) and 48% (10/21), respectively, strongly agreed that they aimed to increase their activity levels over time.

**Conclusions:**

This is the first study to use smartwatch data to report on the 1-year recovery of patients who survived a COVID-19 critical illness. This is also the first study to report on smartwatch use within a post–critical care MDT. Future work could explore the role of smartwatches as part of a randomized controlled trial to assess clinical and economic effectiveness.

**International Registered Report Identifier (IRRID):**

RR2-10.12968/ijtr.2020.0102

## Introduction

Worldwide, the COVID-19 pandemic has resulted in a large cohort of patients presenting to critical care units with acute lung injury requiring protracted ventilatory support. In the United Kingdom alone, from March 2020 to the time of writing, a total of 44,898 patients with confirmed COVID-19 have been admitted to critical care [1].

The patients admitted to intensive care were typically male, were aged over 70 years [2], and spent on average 14 days in critical care [3]. Such patients are at significant risk of postintensive care syndrome [4,5], with studies suggesting that a constellation of physical and psychological problems are likely to persist over a protracted period [6,7].

To date, there is a lack of detailed, long-term outcome data for survivors of COVID-19 critical illness. Furthermore, rehabilitation has been challenged by social distancing, the attenuated availability of health care services, the isolation of survivors from their social support groups, restricted interventions involving face-to-face treatment, and the closure of rehabilitation settings in the community.

Smartwatch use has been rapidly growing, especially over the last 5 years [8]. Smartwatches primarily rely on the pulse wave signal derived from a photoplethysmogram [9] to estimate heart rate. There have been a large number of studies validating wrist-based heart rate measurements in diverse settings [10-14], and several studies have shown that wrist-based wearables provide useful estimates, especially those for resting and low heart rates [15,16]. Fitbit watches use patented photoplethysmogram technology (PurePulse; Fitbit LLC) [17] and have shown reasonable performance and accuracy [12-15,18]. Similarly, Fitbit-estimated, wrist-based step counts have been acceptably accurate in free-living settings, though less so when users exercise vigorously [19]. There is an emerging research base on the health care applications of smartwatches [20], including the surveillance of influenza symptoms [21], the identification of atrial fibrillation [22], chronic airway disease management [23], cardiac rehabilitation [24], and presurgical optimization [25]. In rehabilitation medicine, the use of smartwatch technology provides the possibility of observing the recovery of patients remotely and aiding recovery via detailed, real-time data [26]. Qualitative data suggest that these devices can motivate patients to recover [27]. Although the use of smartwatch devices is evolving and increasing [20], their routine use in this way remains limited.

This study aimed to (1) use smartwatches (Fitbit Charge 3; Fitbit LLC) to monitor changes in the step counts and heart rates of a cohort of participants who survived an admission to critical care during the first wave of the COVID-19 pandemic (April to June 2020) and (2) explore the use of these devices within a rehabilitation setting and report on our experiences with this.

## Methods

### Ethics Approval

Ethical approval was granted by Health Research Authority and Health and Care Research Wales (Yorkshire & The Humber – Bradford Leeds Research Ethics Committee reference number: 20/YH/0157 IRAS 280041).

### Recruitment

#### Study Design and Setting

We conducted a prospective, multicenter observational trial in UK critical care units. The original protocol for this study was published previously [28].

#### Participants

**Figure 1:**
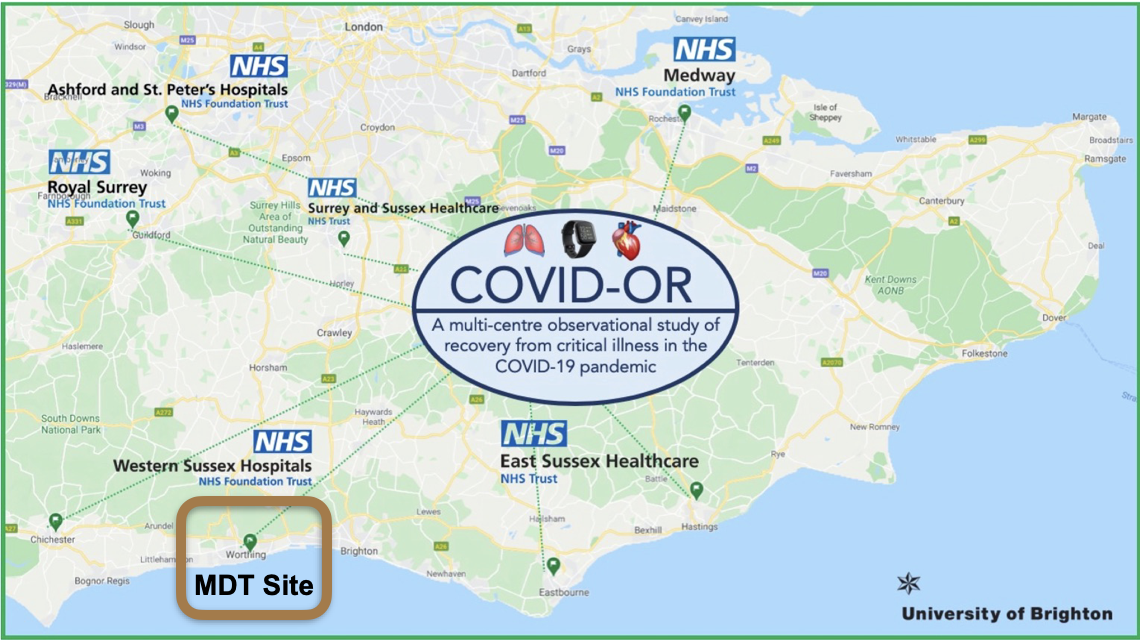
Recruitment sites for COVID-OR. "MDT Site" indicates participants who receive remote monthly support from the multidisciplinary team based on their smartwatch data. All other sites are control sites. COVID-OR: Coronavirus Disease-Observation of Recovery; MDT: multidisciplinary team; NHS: National Health Service.

A total of 50 participants were recruited from 8 UK hospitals in South East England (Figure 1). Adult participants who required invasive positive pressure ventilation or noninvasive ventilation and experienced at least moderate lung injury, which was defined as an arterial oxygen partial pressure to fractional inspired oxygen ratio of ≤26.6 kPa [29], as a result of confirmed COVID-19 were recruited. The exclusion criteria were few and primarily included the lack of a device that was able to host the Fitbit app. The sample size was determined at study inception based on feedback from medical teams in critical care units and based on the current size of their caseloads that met the inclusion criteria and were in line with other feasibility studies of a similar nature [24-27,30].

### Smartwatch Data

At each site, participants were approached at or shortly after discharge from higher dependency care between April and June 2020. Participants were recruited by a physiotherapist or critical care physician. Each site had a local research team for recruiting participants and setting up the smartwatches. Participants were assigned an anonymized study reference ID number, which was used for all data collection procedures. Demographic data were collected for each participant. For the purposes of sample characterization, further data were collected regarding the comorbidities and treatments received during participants’ critical care admission (including the severity of lung injury, the length of stay, and the respiratory support and other organ support received).

Fitbit Charge 3 watches were given to each patient and linked to their anonymized study reference ID numbers. Data were synced to the Fitbit app and then periodically downloaded to a central study database. Participants were asked to wear their smartwatch for as long as they felt able and to ideally aim to use the smartwatch continuously. Participants were given a contact number for a member of the research team that they could use to obtain help for using their smartwatch.

The smartwatch data extracted included daily step count and daily resting heart rate in beats per minute, which was defined as the lowest mean heart rate recorded during a period of inactivity of at least 30 minutes [31]. Further descriptions of the methods via which these smartwatches collect these data are included in Multimedia Appendix 1.

### Smartwatch Usability and Use Within the Multidisciplinary Team

**Figure 2:**
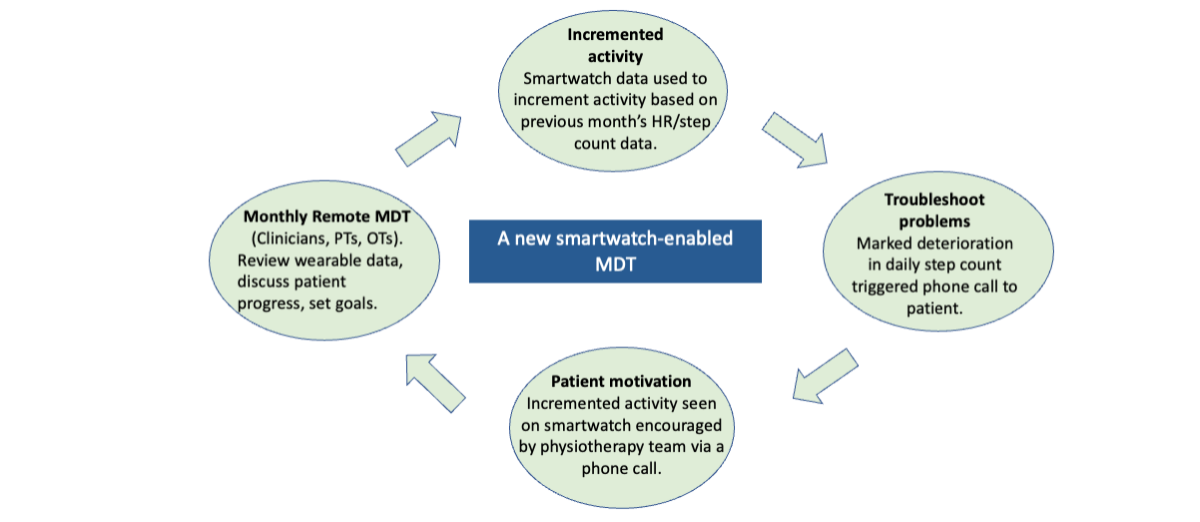
The smartwatch-enabled MDT model. HR: heart rate; MDT: multidisciplinary team; OT: occupational therapist; PT: physiotherapist.

At one site, which was called the *MDT site* (Figure 1), targeted multidisciplinary team (MDT) meetings (including critical care doctors, physiotherapists, occupational therapists, and a nurse) were held monthly for a subset of the overall cohort. These patients had their individual smartwatch data interrogated and reviewed by the MDT each month, and the physiotherapy team used these meetings to determine future exercise plans and rehabilitation goals. A member of the MDT contacted the patients before and after the meetings to inform the MDT about patients. Afterward, feedback was provided to patients with identified issues, and adaptations to rehabilitation plans were agreed on by patients and the MDT (Figure 2). Feedback from members of the MDT was used to assess the feasibility of incorporating smartwatch devices into the post–critical care rehabilitation MDT.

At all other sites (control sites), usual follow-up care was provided without feedback based on the smartwatch data and without MDT intervention.

One-year follow-up visits were completed by a member of the research or physiotherapy team at each site. These were primarily completed via face-to-face or telephone appointments. A patient feedback questionnaire (Multimedia Appendix 2) was completed at this point, and a review of perceptions on using the smartwatches was conducted to assess usability.

### Adherence

Smartwatch use was defined as participants using their watch for a minimum of 1 month. A month was included for analysis if there were over 10 days of data for that month. A data set was considered complete at 1 year if there were data for every month of the year. Patients were considered adherent if they used their smartwatch for any month and were excluded from analysis when there were no data uploaded for any month. Participants were included for comparative analysis at 1 year if data for month 1 and month 12 were present.

### Statistical Analysis

All data were analyzed using R (version 4.0.5; R Foundation for Statistical Computing), and raw data were collected in Microsoft Excel.

*P* values were calculated to determine statistical significance, and actual values were included in analyses unless *P* was <.001. Data were tested for normality via Shapiro-Wilk testing, and significance was tested by using a 2-tailed Student *t* test.

### Patient and Public Involvement

Feedback based on patients’ experiences with the recovery from critical illness was incorporated via patient research champions to inform the design of this study. The COVID-OR (Coronavirus Disease-Observation of Recovery) study steering group had 2 previous patients on the panel that helped to tailor this study to patients’ preferences, and the steering group will help disseminate the results via a patient network of critical care survivors.

## Results

### Sample Characterization

The participants who were recruited across sites in South East England totaled 50. The smartwatch users who were included for analysis totaled 35 participants (MDT site: n=14; control sites: n=21).

For the full cohort, the mean age was 57 (SD 10) years (Table 1), 74% (26/35) of participants were of White ethnicity, and 54% (19/35) had at least 1 comorbidity. The mean length of critical care stay was 18 (SD 16) days, and the mean length of hospital stay was 30 (SD 20) days. There were no statistically significant differences in age (*P*=.22), comorbidities (*P*=.35), and the length of critical care stay (*P*=.37) or hospital stay (*P*=.46) between the MDT and control groups. Similarly, there were no statistically significant demographic differences between smartwatch users (n=35) and nonusers (n=15; Multimedia Appendix 3).

**Table 1:** Demographic comparison of multidisciplinary team (MDT) site–supported participants and control site–supported (all other sites) participants.

Characteristic		MDT site participants (n=14)	Control site participants (n=21)
Age (years), mean (range)		61 (49-73)	57 (35-77)
**Ethnicity, n**			
	White (English, Irish, and any other White background)	9	17
	Asian and Asian British (Indian, Pakistani, Bangladeshi, Chinese, and any other Asian background)	4	3
	Black, African, Caribbean, and Black British (any other Black, African, or Caribbean background)	1	1
**ICD-10^a^ comorbidities, n**			
	None	7	9
	Hypertension	4	7
	Asthma	3	5
Admission weight (kg), mean (range)		84 (53-106)	96 (65-150)
**Length of stay (days), mean (range)**			
	Intensive care unit	17 (5-36)	21 (6-67)
	Hospital	28 (15-49)	33 (10-97)

^a^ICD-10: International Classification of Diseases, Tenth Revision.

### Smartwatch Data

#### Step Count

The full cohort had an average of 4359 (SD 3488) steps per day in the first month following discharge. At 1 year, this had increased to an average of 7914 (SD 4146) steps per day (*P*=.003). Participants had increased their mean step count by 37% (+1630 steps; *P*=.04) from 0 to 3 months following discharge. At 12 months, the mean step count increased by 82% (+3555 steps; *P*=.003) when compared with that for month 0.

**Figure 3:**
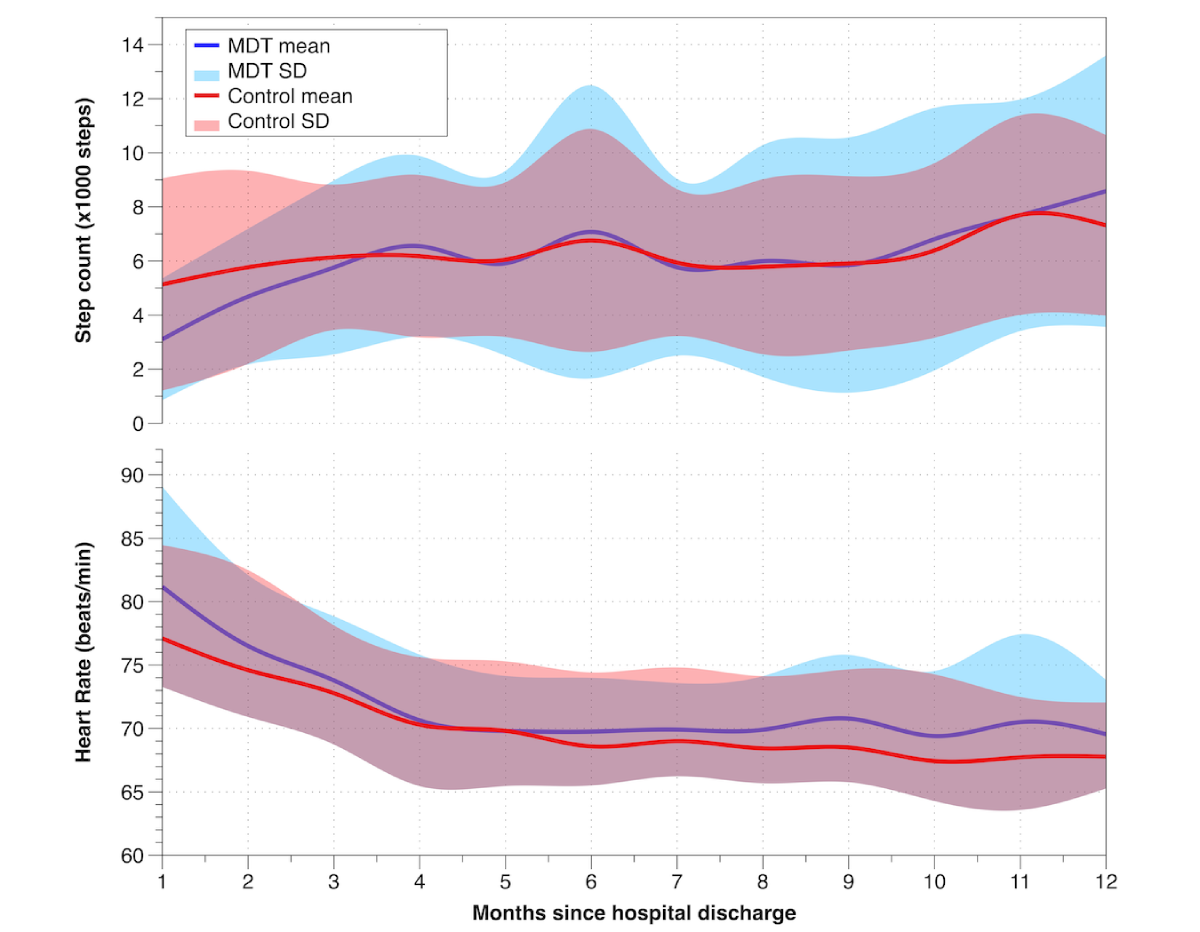
Monthly step counts and heart rates after hospital discharge for MDT-supported participants (blue line: mean; light-blue area: SD) and non-MDT, control site–supported participants (red line: mean; light-red area: SD). MDT: multidisciplinary team.

MDT site participants’ mean step count increased more than that of the control site participants (176% increase vs 42% increase, respectively; +5474 steps vs +2181 steps, respectively; *P*=.04) over 1 year. However, the MDT group was less active than the control site group in the first month (3107 steps vs 5133 steps), and increases were similar between the two groups until month 12 (8581 steps vs 7314 steps; Figure 3).

#### Daily Resting Heart Rate

Heart rates averaged 79 (SD 7) beats per minute in the first month following discharge and 69 (SD 4) beats per minute at 1 year following discharge for the full cohort. Participants had a reduction in mean heart rate of 7% (−6 beats/minute; *P*<.001) at 3 months after data collection and a total reduction in mean heart rate of 13% (−10 beats/minute) by 12 months (*P*<.001; Figure 3). There was no significant difference in heart rate reductions between MDT site (−11 beats/minute; 14% reduction) and control site (−8 beats/minute; 10% reduction) participants over the 1-year period (*P*=.22).

### Smartwatch Usability and Use Within the MDT

**Figure 4:**
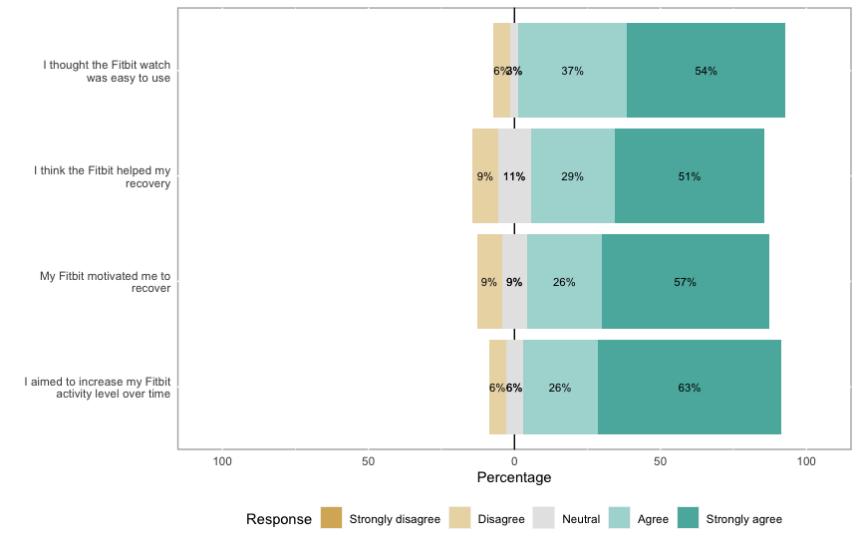
Smartwatch review questionnaire (n=35).

The 1-year review questionnaire revealed that 91% (32/35) of smartwatch users agreed or strongly agreed that their smartwatch was easy to use, 80% (28/35) felt that smartwatches helped them and motivated them to recover, and 83% (29/35) aimed to increase their activity level over time (Figure 4). Participants at the MDT site reported more frequently that they used their smartwatches to help them increase their activity over time (10/14, 71%) and felt that their smartwatch provided more motivation to recover (12/14, 86%) when compared with the control site participants (10/21, 48% and 10/21, 48%, respectively; Multimedia Appendix 4).

In the cohort whose smartwatch data were used to inform the rehabilitation MDT, a sudden reduction in step count among 3 separate participants raised a concern that could be addressed by the MDT. These participants initially received a telephone call to enquire about this reduction in step count. They were then referred to specialist services as required. This prompted the rapid recognition of specific patient problems prior to the patients self-reporting the problem to a clinician. Examples of such problems include acute joint inflammation and myocardial ischemia. Further feedback from the MDT members suggested that participants in the MDT subgroup felt supported and reassured by the observations of the clinical team, and this positively improved participants’ recovery.

### Adherence

The adherence rates for smartwatch use were 70% (35/50) in the overall cohort, 74% (14/19) in the MDT group, and 68% (21/31) at the control site (Table 2). Of the 35 participants included for analysis, 12 (34%) had a complete data set with activity data and heart rate recorded for every month of the 1-year period. Further, 25 patients had data for the first and last months of this study. For the 23 participants with incomplete data sets, the average number of months with data was 7 for both step count and heart rate. Additionally, 2 watches failed and were returned to the manufacturer, and 1 watch strap broke, which rendered a user unable to wear their watch until another was provided.

**Table 2:** Comparison of smartwatch users and nonusers by site.

Characteristic	Overall cohort (N=50)	Multidisciplinary team site participants (n=19)	Control site participants (n=31)
No smartwatch use (excluded from analysis), n (%)	15 (30)	5 (26)	10 (32)
Adherent to smartwatch use, n (%)	35 (70)	14 (74)	21 (68)
Complete data set at 1 year, n (%)	12 (24)	4 (21)	8 (26)

## Discussion

### Principal Findings

This multicenter study demonstrated that smartwatches can be used to observe a significant increase in participants’ daily step counts over a 12-month recovery period from COVID-19–induced critical illness. This study also provides information on the use of a remote critical care rehabilitation MDT that used smartwatch data to support patient recovery. Smartwatches were perceived to be user-friendly, were well tolerated, and added value by providing rapid feedback to the MDT. On 3 occasions, the smartwatch data provided actionable data to the MDT that triggered referrals to other specialties.

Participants were discharged from hospitals after critical care admissions and significant deconditioning, and step counts were well below those of active adults (8000 to 10,000 steps per day) [32,33] and those found to be associated with a decreased risk of all-cause mortality [34]. Capturing this trajectory of improvement via the smartwatches provided data that suggest physiological recovery and are reassuring for patients with severe illness.

This study demonstrated that smartwatches can allow monitoring of physical activity remotely, though a considerable number of participants, despite perceiving their devices to be easy to use, did not use them regularly. This presents a limitation to this study but also adds important information to critical care rehabilitation literature, and future studies might need to include a similar dropout rate when using smartwatches in a similar cohort or assessing a smartwatch intervention. Our smartwatch usage rates are broadly similar to those of previous studies [35,36], though there were no identified studies with a similar cohort that allowed for direct comparisons of use.

Device use was similar between the MDT site and control sites and suggested that some participants were not motivated to use their smartwatch despite regular reminders from a member of the MDT. The reasons for inadequate data included the infrequent use of the smartwatch; hardware failure; the failure to sync data despite participants using the watch; and lastly, participants not wearing the device at night.

Although the quantitative results suggested limited differences between the MDT subgroup and control groups, the feedback from participant feedback questionnaires suggested an increased perception that the smartwatches provided motivation for recovery and for increasing activity levels over time in the MDT site group. Further, while we acknowledge that recovery is a complex phenomenon and that, similarly, an MDT is a complex intervention, these responses might provide insight into an intervention group that could be encouraged to become fitter via the use of smartwatches.

### Comparison With Prior Work

Although the use of smartwatches is increasing [20] and the adoption of digital technology during the COVID-19 pandemic has become widespread [37], many related studies adopt technology for diagnosis [38], surveillance [39], and the prevention of disease, with few targeting technology for rehabilitation, empowerment, or patients’ engagement with rehabilitation. To our knowledge, this is the first study of its kind to use smartwatches in this way for COVID-19 survivors. One study [40], which is in the early recruitment phase, is looking to evaluate the feasibility of delivering a remotely monitored rehabilitation program for critical care survivors with COVID-19.

There are limited reports of 1-year outcome data for post–critical care survivors with COVID-19. However, data from 1-year outcome studies are in line with our data, and such studies have reported significant recovery from COVID-19 illness [41], albeit in survivors who vary widely in terms of disease severity.

### Strengths and Limitations

First, the resource limitations involved in recruitment during the first wave of a global pandemic resulted in little data being available regarding the number of patients who were initially approached but declined to participate in this study. Centers approached as many participants as their resources allowed, and despite our demographic data suggesting that our participant samples were representative of the critical care population at the time, the little data we have regarding the number of initially approached participants and those who declined to participate may limit the generalizability of our results.

Second, in a multicenter observational study using wearable technology during a pandemic, missing data are inevitable. The management of these missing data was challenging and was carefully considered. We believe that our data, which were collected for at least 10 days in a given month, were representative of the sample, especially given the high sampling frequency (amount of data collected per minute) of the smartwatches.

### Future Directions

This study explored the use of a smartwatch-enabled MDT, and the next steps should be to robustly assess clinical effectiveness and cost-effectiveness in an adequately powered randomized controlled trial. The analysis of patients’ perceived recovery and smartwatch-assessed activity levels in a larger study would also provide further insight into the use of smartwatch devices. Overall, this smartwatch-assisted approach could lend itself to other clinical contexts where physical optimization is crucial, such as perioperative settings for those undergoing major surgery.

### Conclusion

Smartwatches can be used to observe an increase in activity among patients following hospital admission with COVID-19 critical illness. The observed trend in daily step counts was encouraging, given the severity of the illness and the level of deconditioning at hospital discharge. Though a considerable number of participants did not use their smartwatches as intended, the technology was used to support the care delivered to participants in a remote MDT setting and was able to detect significant changes in activity levels. Further work is required to assess the clinical effectiveness and cost-effectiveness of this intervention and whether it can result in improved patient outcomes and quality of life.

## Appendix

Multimedia Appendix 1 Further smartwatch data acquisition information.

Multimedia Appendix 2 Smartwatch assessment questionnaire.

Multimedia Appendix 3 Demographic comparison of users versus nonusers of smartwatches.

Multimedia Appendix 4 Smartwatch response questionnaire by site.

## Abbreviations

BASEM: British Association of Sports and Exercise Medicine
COVID-OR: Coronavirus Disease-Observation of Recovery
MDT: multidisciplinary team
NHS: National Health Service

## Data Availability

Smartwatch data are available on request from the lead author.
