# Supplementary material for: Using Smartwatches to Observe Changes in Activity During Recovery From Critical Illness Following COVID-19 Critical Care Admission: 1-Year, Multicenter Observational Study
Source: JMIR Rehabil Assist Technol. 2022 May 2;9(2):e25494. doi: 10.2196/25494 (PMC9063865; doi:10.2196/25494)
Supplement: Multimedia Appendix 1 [file rehab_v9i2e25494_app1.docx]

## Multimedia Appendix 1

### Further smartwatch data acquisition information

All participants were set up with smartwatches (Device manufacturer: Fitbit, Device type: Charge 3, Device version: 3rd, Firmware version: version 1.88.11, Hardware: Sampling rate 1 sec during exercise, 5s all other times.)[16]. The smartwatches were set up by a member of the study team with written information given to the patient regarding device use. Smartwatches were linked to the unique study ID email address which in turn were linked to a Fitbit account. Study participants were given a copy of their study ID email and password and used this to login into the Fitbit application, installed on their phone or tablet. The watch uploaded (‘synced’) data to the Fitbit app automatically. This was then periodically downloaded to a central database.

### Daily resting heart rate (HR) in beats per minute (bpm).

The Fitbit device uses an accelerometer to determine that the user is not moving and the optical signal is estimated to be a good quality. Where the users wear their device at night, this data is included by Fitbit into the resting heart rate calculation. In order to have sufficient data, a person must have at least 30 minutes of still periods with valid data during the day for a DRHR to be reported [31].

### Daily step count.

Fitbit devices use a microelectronic 3-axis accelerometer to capture body motion in 3-dimensional space. This data is analysed using proprietary algorithms to identify patterns of motion to identify daily steps taken, energy expenditure, sleep, distance covered, and time spent in different intensity of activities.
