# Supplementary material for: Using Smartwatches to Observe Changes in Activity During Recovery From Critical Illness Following COVID-19 Critical Care Admission: 1-Year, Multicenter Observational Study
Source: JMIR Rehabil Assist Technol. 2022 May 2;9(2):e25494. doi: 10.2196/25494 (PMC9063865; doi:10.2196/25494)
Supplement: Multimedia Appendix 2 [file rehab_v9i2e25494_app2.docx]

## Multimedia Appendix 2

### Smartwatch assessment questionnaire

Asked at face to face 1 year review to participants by a member of the research team

Regarding my fitbit device:

1) I thought the fitbit watch was easy to use

Strongly agree-Agree-Neutral-Disagree-Strongly disagree

2) I think the fitbit helped my recovery

Strongly agree-Agree-Neutral-Disagree-Strongly disagree

3) My fitbit motivated me to recover

Strongly agree-Agree-Neutral-Disagree-Strongly disagree

4) I thought the Fitbit app on my phone was easy to use

Strongly agree-Agree-Neutral-Disagree-Strongly disagree

5) I used the information from my Fitbit to monitor my activity levels

Strongly agree-Agree-Neutral-Disagree-Strongly disagree

6) I aimed to increase my Fitbit activity level over time.

Strongly agree-Agree-Neutral-Disagree-Strongly disagree

7) I wore my Fitbit watch

Every day- Most days- Weekly- Monthly- Never

8) I reviewed the information from my Fitbit

Every day- Most days- Weekly- Monthly- Never
