# Supplementary material for: Using Smartwatches to Observe Changes in Activity During Recovery From Critical Illness Following COVID-19 Critical Care Admission: 1-Year, Multicenter Observational Study
Source: JMIR Rehabil Assist Technol. 2022 May 2;9(2):e25494. doi: 10.2196/25494 (PMC9063865; doi:10.2196/25494)
Supplement: Multimedia Appendix 3 [file rehab_v9i2e25494_app3.docx]

## Multimedia Appendix 3

#### Demographic comparison of users vs non-users of smartwatches

|  | Users | Non Users | *P* value |
| --- | --- | --- | --- |
| **Number** (n) | 35 | 15 |  |
| **Age** (mean,SD) | 59(10) | 55(10) | 0.24 |
| **Ethnicity (n)** |  |  |  |
| *White (English, Irish, any other white background)* | 26 | 12 |  |
| *Asian / Asian British (Indian, Pakistani, Bangladeshi, Chinese, any other asian background)* | 7 | 1 |  |
| *Black / African / Caribbean / Black British (any other Black/African/Carribean)* | 2 | 1 |  |
| *Mixed / Multiple Ethnic (White and black carribean, White and Black African, white and asian)* | 0 | 1 |  |
| **ICD-10 comorbidities (n)** |  |  |  |
| None | 16 | 4 |  |
| Hypertension | 12 | 7 |  |
| Asthma | 6 | 1 |  |
| Diabetes | 0 | 1 |  |
| Admission weight (kg) (mean,SD) | 91(20) | 96(12) | 0.41 |
| **Length of stay (LOS) (days)** |  |  |  |
| ICU LOS (mean,SD) | 19(15) | 25(18) | 0.22 |
| Hospital LOS (mean, SD) | 31(21) | 40(22) | 0.11 |
|  |  |  |  |
