# Supplementary material for: Using Smartwatches to Observe Changes in Activity During Recovery From Critical Illness Following COVID-19 Critical Care Admission: 1-Year, Multicenter Observational Study
Source: JMIR Rehabil Assist Technol. 2022 May 2;9(2):e25494. doi: 10.2196/25494 (PMC9063865; doi:10.2196/25494)

## Multimedia appendix 4

### Smartwatch response questionnaire by site

1. Smartwatch response questionnaire; MDT Site (n=14)


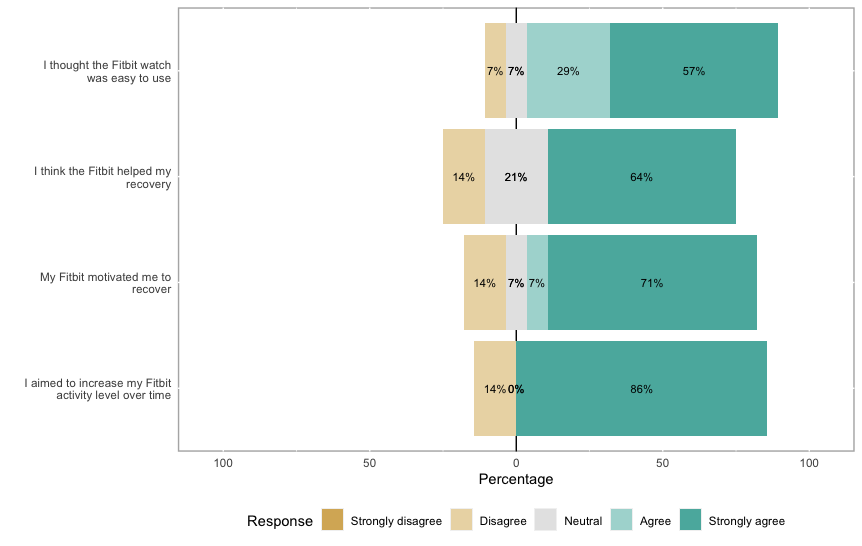


1. Smartwatch response questionnaire; Control Site (n=21)


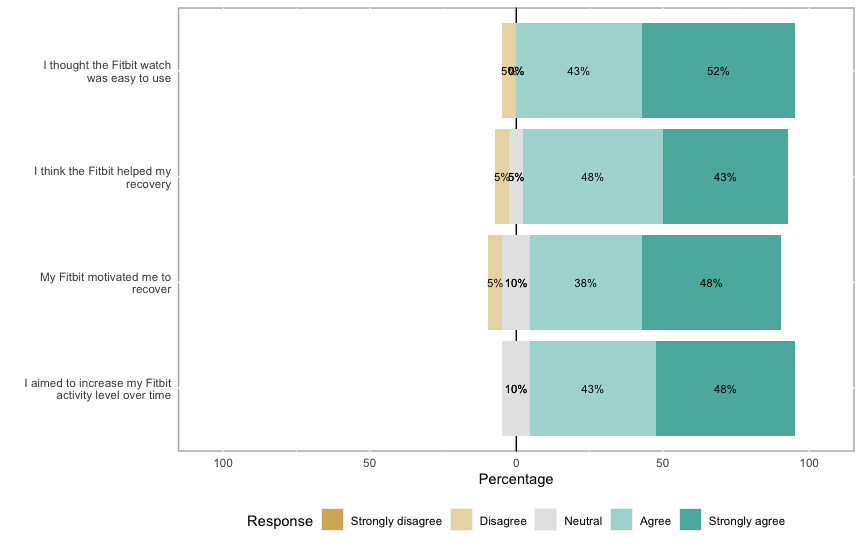

Supplement: Multimedia Appendix 4 [file rehab_v9i2e25494_app4.docx]
